# Family Functioning and Parenting Styles in Adolescents Diagnosed with Trichotillomania

**DOI:** 10.1007/s10578-025-01811-9

**Published:** 2025-02-04

**Authors:** Hande Günal Okumuş, Meryem Kaşak, Yusuf Selman Çelik, Ayşe Selma Yenen Menderes, İsranur Yenen Sivri, Rahime Duygu Temeltürk, Devrim Akdemir

**Affiliations:** 1 Department of Child and Adolescent Psychiatry, Uşak Training and Research Hospital, Uşak, Türkiye; 2 Department of Child and Adolescent Psychiatry, Ankara Etlik City Hospital, Ankara, Türkiye; 3 Department of Child and Adolescent Psychiatry, Ankara Atatürk Sanatoryum Training and Research Hospital, Ankara, Türkiye; 4 Department of Child and Adolescent Psychiatry, Osmaniye State Hospital, Osmaniye, Türkiye; 5 Department of Child and Adolescent Psychiatry, Ankara University Faculty of Medicine, Ankara, Türkiye; 6 Department of Interdisciplinary Neuroscience, Ankara University Institute of Health Scienves, Ankara, Türkiye; 7 Department of Child and Adolescent Psychiatry, Hacettepe University Faculty of Medicine, Ankara, Türkiye

**Keywords:** Trichotillomania, Family functioning, Parental styles, Adolescent

## Abstract

This study aimed to compare the family functioning and parenting styles in adolescents with trichotillomania (TTM) and healthy controls and to assess the relationships between the family functioning, parenting styles, and clinical features of TTM, including the severity of TTM, duration of illness, and concomitant psychiatric symptoms. The study sample consisted of 100 adolescents aged 12–18 years, 50 patients with TTM, and 50 healthy controls. All participants completed a sociodemographic and clinical data form, the Massachusetts General Hospital Hairpulling Scale (MGH-HPS), the Revised Children’s Anxiety and Depression Scale-Child Version (RCADS-CV), the Family Assessment Device (FAD), and the Perceived Parental Attitude Scale (PPAS). Results show that adolescents with TTM reported higher levels of comorbid anxiety and depression symptoms than healthy controls, and 64% of adolescents with TTM had at least one comorbid psychiatric disorder. Parents of adolescents with TTM also reported more significant impairment in the affective responsiveness, problem-solving, and general family functioning subscales of the FAD. Adolescents with TTM endorsed significantly more authoritarian perceptions of both parents on the PPAS. While the severity of hairpulling symptoms showed only a positive and significant relationship with the RCADS-CV total internalizing score, the duration of illness was positively and moderately correlated with the impairment in affective responsiveness, problem-solving, and general family functioning subscales of FAD. Finally, the RCADS-CV total internalizing score and the general functioning subscale of FAD were the most important predictors of TTM. The implications of family functioning and parenting styles, future targets for family-focused approaches in TTM, and study limitations are discussed.

## Introduction

Trichotillomania (TTM) is a psychiatric disorder characterized by repetitive hair-pulling behaviour that leads to hair loss without an underlying dermatological condition [1]. TTM usually begins in childhood with a bimodal distribution, peaking in early childhood (2 to 6 years) and early adolescence (10 to 13 years) [2, 3]. Many studies have shown that early-onset TTM is associated with less severity, a lower prevalence of psychiatric comorbidity, and a better treatment outcome than later-onset TTM [4–6]. Our current knowledge about the prevalence of TTM among children and adolescents is based mainly on small-scale and clinically oriented research, with a lifetime prevalence of approximately 1% [7, 8]. Although most studies have found higher prevalence rates in females than in males in adolescents [9, 10], some studies have found more equal gender distribution rates, particularly in early childhood [8, 11]. Despite its high prevalence and significant impact on physical, social, emotional, academic, and family functioning, relatively little is known about the etiology and associated risk factors for TTM.

Although there is no specific definition of healthy family functioning in the existing literature, it refers to effective and transparent communication, mutual appreciation and encouragement, emotional cohesion between family members, the presence of specific rules and roles within the family structure, the ability to cope with crises, and caring for each other such as being attentive and responsive to each other’s needs [12, 13]. Impaired family functioning, which plays a central role in the holistic physical, socio-emotional, and mental development of children, is not only a significant risk factor for developing childhood psychopathologies but also has a profound impact on adult mental health during the transition into adulthood [14]. Although family functioning has also been identified as a potential area of research that could contribute to a better understanding of the etiology and treatment of TTM, studies assessing family functioning in pediatric TTM are scarce. In an early study, Reeve et al. (1992) assessed family functioning in 10 cases of individuals aged 6 to 17 years diagnosed with TTM using the Family Environment Scale (FES) [15] in a cross-sectional design with no control comparison group. They reported that youths with TTM characterized their parents as “less cohesive, less emotionally expressive, and more likely to restrict autonomy” [16]. In another study of 50 adolescents with TTM, 23 matched healthy controls. For the parents of both groups, a comprehensive analysis was conducted to assess family environment variables and parental stress using the FES, Family Assessment Measure, Version III (FAM-III) [17] and the Stress Index for Parents of Adolescents (SIPA) [18] in a cross-sectional design. They found that adolescents with TTM reported higher levels of impaired family functioning, particularly in terms of more anger expression, aggression, conflict, and lower levels of family support, and that overall parental stress was higher than controls [19]. Consistent with previous research, a recent study assessing family functioning and perception of parenting behavior using the FES and the Children’s Report of Parenting Behavior Inventory (CRPBI) [20] in adolescents with obsessive-compulsive disorder (OCD) (n = 30), TTM (n = 30) and 30 matched healthy controls found that both adolescents and their parents in the TTM group reported lower emotional expressiveness and cohesion [21]. Given the central role of parents in ensuring adolescents’ adherence to treatment and maintaining therapeutic progress and the bidirectional relationship between TTM and family dysfunction, the literature often emphasizes the importance of assessing family functioning when developing therapeutic interventions [22, 23].

Parenting styles can be defined as directly observable and common strategies that parents use in raising their children. It significantly impacts the formation of a positive self-image, the development of adaptive emotional regulation and problem-solving skills, and the establishment of robust interpersonal relationships in childhood [24]. Baumrind, who is considered to be a pioneer of research into parenting styles, originally defined three distinct parenting styles: *authoritarian*, *authoritative* (also known as *democratic*), and *permissive* [25]. An authoritarian parenting style has been consistently associated with negative outcomes in youth, such as aggression, depression, and anxiety [26–29]. In particular, high parental control, low independence, and enhanced responsibility are often reported in the families of youths with OCD [22, 30]. Although many studies have investigated the effects of negative parenting styles on child psychopathology, there are some limitations to the existing literature. The most important of these is that most of the studies are cross- sectional. Another major limitation is that they have been based on maternal data only, and very few studies have explicitly examined the effects of paternal attitudes and used heterogeneous samples in terms of gender without controlling for the confounding effect of gender [31, 32]. While parenting styles have been examined in many childhood psychopathologies, to the best of our knowledge, only one study has investigated parenting styles in children and adolescents with TTM. Peris et al. (2019) assessed family functioning and parenting style using the FES and CRBPI. Although the TTM group reported more impaired family functioning on the FES compared to the other groups, there was no statistically significant difference between the TTM, OCD, and healthy controls in terms of parenting styles. In addition, no significant relationship was found between the severity of TTM and parenting styles [21]. However, the discussion section of the article, while focusing on the issue of greater impairment in family functioning in the TTM group compared to the other groups, does not address possible reasons for the lack of significant differences in parenting styles between the groups. Therefore, the primary aim of this study is to compare the family functioning and maternal and paternal parenting styles in adolescents with TTM with healthy controls. The secondary aim is to assess the relationships between family functioning, parenting styles, concomitant anxiety/depressive symptoms and clinical features of TTM, including the severity of TTM and duration of illness. Moreover, the last aim of the study is to investigate the predictors of TTM in adolescent patients.

We hypothesized that family functioning would be more impaired and perceptions of both parenting styles would be more authoritarian in the TTM group, as assessed by self-report scales. In addition, we hypothesized that impaired family functioning, authoritarian parenting styles and concomitant anxiety/depressive symptoms would be positively associated with the severity of TTM and duration of illness. Finally, advanced age, female gender, low socio-economic status, impaired family functioning and authoritarian parenting styles (for both parents) would be important predictors of TTM in adolescents.

## Method

### Participants

This multicenter study was conducted with a sample of 100 adolescents and their primary caregiver(s) in five hospitals within three geographical regions. These hospitals were Uşak Research and Training Hospital (Aegean region), Ankara Etlik City Hospital, Ankara Atatürk Sanatoryum Research and Training Hospital, Ankara University Faculty of Medicine (Central Anatolia region), and Osmaniye State Hospital (Mediterranean region). Inclusion criteria for the TTM group were as follows: (1) age between 12 and 18 years, (2) current diagnosis of TTM according to DSM-5, (3) availability of at least one biological parent to participate in the study, (4) willingness to participate in the study, and complete data on all relevant measures (For two-parent families, a semi-structured interview was conducted with the adolescents and both parents. The scale administered to the family was completed by both parents together). Adolescents diagnosed with autism spectrum disorders, psychotic disorders, bipolar disorder, alcohol/substance-related disorders, intellectual disability, language disorders, or any chronic medical condition were excluded. The exclusion criteria for the parents were being not literate, unwillingness to participate, and having diagnoses of intellectual disability or psychotic disorder. Two patients with a comorbid diagnosis of intellectual disability, three patients with subthreshold symptoms (failure to meet significant impairment criteria for TTM) and two patients whose parents declined to participate were subsequently excluded from the TTM group.

The healthy control group consisted of age- and gender-matched adolescents who volunteered to participate in the study did not meet the diagnostic criteria for any psychiatric diagnosis, and had no history of mental health problems or medication. Three adolescents who were initially assessed for eligibility in the healthy control group were subsequently excluded from the study because two of them were diagnosed with a psychiatric disorder, and the other’s family environment measure was missing. Participants (*n* = 10) who did not meet the inclusion criteria for either group were excluded from the present analyses. Ultimately, 50 adolescents (44 female, six male) with TTM and their parents and 50 healthy controls (45 female, five male) were included.

The ethical committee of the Usak University approved the protocol (No:344-344-02). Comprehensive verbal information was provided about the methodology, time schedule, what would be expected of the participants, potential benefits/risks, and confidentiality of the data. Written informed consent was also obtained from all the adolescents and their parents.

### Procedures

Following the approval of the ethical committee, the records of patients who presented to the hospitals between 2021 and 2023 who were diagnosed with “Trichotillomania, ICD code F63.3” on the basis of a on psychiatric assessment were requested from the information processing departments of the hospitals. The families of these patients were contacted using the telephone number in the electronic health records. Information about the study was provided and, for those who agreed to participate, appointments were made at specified times and dates, after which interviews were conducted with both the patients and their families. In addition, adolescents who presented to the child and adolescent mental health clinics for the first time between February and July 2024 and were diagnosed with TTM following a psychiatric assessment were also included in the study.

The healthy control group were selected from adolescents who had applied to these clinics for psychiatric counselling (seeking guidance on managing the developmental characteristics of adolescence or seeking professional counseling due to an acute crisis situations, such as family conflicts or difficulties in peer and romantic relationships etc.). They were screened via semi-structured psychiatric interviews to ensure that they did not meet the diagnostic criteria for any psychiatric disorder. Self-report scales were also administered, and comprehensive information about their academic performance and social adjustment was gathered from school counselors and classroom teachers. These assessments were conducted between February and July 2024, in line with the patient recruitment period.

### Measures

A sociodemographic and clinical data form was developed by the researchers to assess the sociodemographic variables of both the research and healthy control groups, such as age, gender, socioeconomic status (SES) classified according to the Hollingshead-Redlich Scale (HRS), family structure, and clinical information relevant to TTM, including the duration of symptoms, comorbid psychiatric diagnoses, and use of pharmacological agents. A sociodemographic and clinical data form was completed by the researchers following the interview with the participants.

***The Hollingshead-Redlich Scale (HRS)*** was first developed by Hollingshead and Redlich in 1958 to assess the socioeconomic level of families [33]. It assigns scores based on the parents’ highest educational and occupational level, considering the occupations and educational backgrounds of both the mother and father. Classification is on a scale of 1 to 5, with lower scores indicating higher SES. Given the distributional characteristics of our sample, this study examined SES in three categories: low (4–5), middle (3), and high (1–2). The Hollingshead-Redlich scale was rated by the researchers.

***The Kiddie-Schedule for Affective Disorders and Schizophrenia for School-Age Children-Present and Lifetime Version DSM-5***,*** K-SADS-PL***, is a semi-structured interview developed initially by Kaufman et al. to assess current and lifetime psychopathologies in children and adolescents [34]. It has been adapted to the Turkish population according to the DSM-5 criteria [35]. The K-SADS-PL was administered to the adolescents and their parents by a child and adolescent psychiatrist to identify comorbid psychiatric disorders in the TTM group and to exclude adolescents with psychiatric disorders in the healthy control group. The K-SADS-PL was administered to the adolescents and their parents by the certified child and adolescent psychiatrists to identify comorbid psychiatric disorders in the TTM group, and to ensure the absence of any psychiatric diagnoses in the healthy control group.

***The Massachusetts General Hospital Hairpulling Scale (MGH-HPS)*** is a 7-item self-report measurement to assess the severity of TTM, including frequency, intensity, and control of hair pulling, as well as distress associated with hair pulling over the past week [36]. Each item is rated on a scale from 0 to 4, with higher scores reflecting greater severity. MGH-HPS is a reliable and valid measurement tool for TTM in Turkish samples [37]. In this study, Cronbach’s alpha was 0.789. This form was completed by the adolescents in the TTM group.

***The Revised Children’s Anxiety and Depression Scale-Child Version (RCADS-CV****)*, which was developed by Chorpita et al. (2005), is a self-report questionnaire designed to assess dimensions of DSM-IV-based anxiety and depressive disorders in children and adolescents using a 3-point scale [38]. The RCADS-CV consists of 47 items in six subscales: generalized anxiety disorder (GAD, six items), OCD (six items), social anxiety disorder (SAD, seven items), separation anxiety disorder (SPD, nine items), panic disorder (PD, nine items), and major depressive disorder (MDD, ten items). The total scale ranges from 0 to 141. The raw scores of the subscales were calculated and scored using the corresponding T-score. A T-score of 65 or more is considered clinically significant. The reliability and validity of the Turkish version of the RCADS-CV have been demonstrated in children and adolescents aged between 8 and 17 years [39]. In the present study, Cronbach’s alpha was 0.961. The Cronbach’s alpha coefficients for the subscales were as follows: 0.893 for SAD, 0.741 for SPD, 0.844 for OCD, 0.886 for PD, 0.786 for GAD, and 0.917 for MDD. This form was completed by the adolescents.

***The Family Assessment Device (FAD)*-*Parent Version*** was first developed in 1983 by clinically applying the McMaster Family Functions Model to families to assess family functioning [40]. The FAD consists of 47 items in seven subscales: problem-solving (six items), communication (nine items), roles (eleven items), affective responsiveness (six items), affective involvement (seven items), behaviour control (nine items), and general functioning (twelve items). The scores obtained are summed for each sub-dimension and then averaged. “Two” is the cut-off value, and mean scores above two indicate impaired family functioning. The validity and reliability of the Turkish version of the FAD have been demonstrated in Turkish samples [41]. In the present study, the Cronbach’s alpha was 0.91. The Cronbach’s alpha coefficients for the subscales were as follows: 0.787 for problem-solving, 0.693 for communication, 0.665 for roles, 0.728 for affective responsiveness, 0.196 for affective involvement, 0.477 for behavior control and 0.834 for general functioning. This form was completed by the parents.

***The Perceived Parental Attitudes Scale (PPAS)***, revised by Bilal [42] based on Kuzgun’s PPAS [43], is a 50-item self-report questionnaire designed to assess perceived parental attitudes. The PPAS has two distinct subscales: democratic (25 items) and authoritarian attitudes (25 items). The PPAS generates separate scores for maternal and paternal attitudes, with lower scores indicating authoritarian attitudes. Examples of items representing democratic attitudes include: *She/he takes my wishes and needs into account when making decisions about me*; *She / he has always shown sensitivity toward my feelings and needs; She/he supports me in developing a healthy perspective on issues related to sexuality; She/he encourages social engagement by allowing me to invite friends to our home and to participate in meetings with my peers.* Examples of items representing authoritarian attitudes include: *She/he claims that a “good child” is one who grows up according to their parents’ expectations and strictly follows their instructions; She/he considers corporal punishment as a “divine gift” and does not hesitate to use it when she/he deemed it necessary; She/he strictly forbids me to associate with people of whom he/she disapproves*,* She/he punishes even my small mistakes severely.* In the present study, the Cronbach’s alpha coefficient of the PPAS for the total scale was 0.70. The Cronbach’s alpha coefficients for the subscales were as follows: 0.943 for maternal democratic, 0.960 for paternal democratic, 0.893 for maternal authoritarian and 0.898 for paternal authoritarian. This form was completed by the adolescents.

### Data Analyses

Statistical Package for the Social Sciences (SPSS) version 26.0 was used to analyze the data. The suitability of the variables for normal distribution was tested using visual (histogram and probability plots) and analytical methods (Kolmogorov-Smirnov/Shapiro-Wilk tests). Descriptive statistics are shown as mean ± standard deviation for normally distributed variables, as median (minimum-maximum) for non-normally distributed variables, and as number of cases and percentage (%) for categorical variables. The t-test was used when parametric test assumptions were met, and the Mann–Whitney U test was used when parametric test assumptions were not met. The chi-square test or Fisher’s exact test was used to compare the categorical data of the two groups. Pearson’s or Spearman’s coefficients were used to assess the linear association between two variables. Three binary logistic regression analyses were performed to concurrently examine the relationships between family characteristic variables (FAD_general functioning, PPAS_M authoritarian, PPAS_P authoritarian) and potential covariates (age, gender, SES, and total anxiety and depressive symptom/internalizing scores) on the presence of TTM. Goodness of fit was measured using the Hosmer-Lemeshow test (*p* >.05 was considered adequate), and global predictive ability was measured using Nagelkerke’s pseudo-R2 coefficient.

## Results

### Sociodemographic and Clinical Characteristics

Table 1 shows the sociodemographic characteristics of the TTM and healthy control groups. The median age of the adolescents in both groups was 15 years [median: 15 (12–18)]. There were no significant differences in age, gender, and family structure between the two groups. The SES was significantly lower in the TTM group than in the healthy controls.

The mean score on the MGH-HPS scale in the research group was 16.9 ± 4.43. The mean duration of the TTM symptoms was 2.7 years [median: 2.3 (0.2-7.0)].

Of the TTM patients, 18 (36%) had no comorbiditiy, and 32 (64%) had at least one comorbidity [eleven patients with GAD, nine patients with MDD, four patients with ADHD- combined type, three patients with ADHD- inattentive type, one patient with ADHD-hyperactive type, six patients with SAD, one patient with SPD, one patient with OCD, one patient with Specific Phobia (SP) and one patient with Persistent Depressive Disorder (PDD)]. It was noted that ten patients (20%) were undergoing pharmacological agents in the TTM group (five patients with sertraline, two patients with risperidone, one patient with aripiprazole, one patient with atomoxetine and one patient with methylphenidate).

**Table 1:** Groups’ sociodemographic characteristics

	TTM	Control	Test statistics	*p*
Age, median (min-max)	15(12–18)	15(12–18)	Z =.258^a^	0.797
Gender (%) Female Male	44(88) 6(12)	45(90) 5(10)	χ2 =.102^b^	0.749
SES (%)				
High^*^	15(30)	29(58)		
Middle	20(40)	11(22)	χ2 = 8.067^b^	0.018^*^
Low	15(30)	10(20)		
Family structure (%)				
Nuclear	43(86)	44(88)		
Extended	1(2)	4(8)		
Divorce	3(6)	1(2)	χ2 = 4.811^b^	0.307
Step parent	1(2)	1(2)		
Death of one of the parents	2(4)	0(0)		

*TTM* trichotillomania, *SES* socioeconomic status

^a^: Mann–Whitney U test

^b^: Chi-square test

^*^p <.05

### A Comparison of the Groups’ Internalizing Symptoms, Family Functioning, and Parenting Styles

Table 2 shows both groups’ self-report measurement scores. The TTM and healthy control groups were compared in terms of RCADS-CV, FAD and PPAS total scores and subscale scores. All subscale scores on the RCADS-CV differed significantly between the two groups and were significantly higher in the research group (*p* <.001 for all). Problem-solving, affective responsiveness, and general functioning subscales of the FAD were significantly higher in the TTM group than in the healthy controls, pointing out more impaired family functioning. In the TTM group, the scores for the democratic attitudes of both parents were found to be significantly lower, whereas the scores for the authoritarian attitudes of both parents were found to be significantly higher than those of the control group.

**Table 2:** Comparison of self-report measurement scores between the TTM and control groups

	TTM	Control			
	Median (min-max)	Median (min-max)	Z	*p*	Effect size (*r*)
**RCADS-CV**					
SAD	63,5(30–80)	44(24–60)	6,413	< 0.001^***^	0.641
PD	73(40–80)	54(37–80)	5,524	< 0.001^***^	0.552
MDD	71(37–80)	46(31–71)	6,473	< 0.001^***^	0.647
SPD	65(38–80)	48(38–80)	4,636	< 0.001^***^	0.463
GAD	61(36–80)	46(33–64)	5,932	< 0.001^***^	0.593
OCD	67(38–80)	48(32–71)	5,779	< 0.001^***^	0.577
Total anxiety	69,5(33–80)	48(30–65)	6,996	< 0.001^***^	0.699
Total internalizing	69(35–80)	47.5(29–65)	7.131	< 0.001^***^	0.713
**FAD**					
Problem solving	2(1-3.3)	1.7(1-3.5)	2.647	0.008^**^	0.264
Communication	1.9(1-3.3)	1.7(1-2.8)	1.576	0.115	
Roles	2.1(1.3–3.2)	1.9(1.3-3)	1.808	0.071	
Affective responsiveness	1.5(1-3.5)	1.4(1-2.7)	2.202	0.028^*^	0.264
Affective involvement	2.4(1.7–3.6)	2.3(1.9–3.3)	1.048	0.294	
Behavior control	2.1(1.3-3)	1.9(1.3–3.1)	0.785	0.432	
General functioning	1.8(1-3.2)	1.5(1-2.7)	2.957	0.003^**^	0.295
**PPAS**					
PPAS_M democratic	93(30–123)	106(56–125)	-2.783	0.005^**^	-0.278
PPAS_M authoritarian	64(31–105)	51.5(26–97)	3.879	.<001^***^	0.387
PPAS_P democratic	80.5(28–117)	98(32–125)	-3.069	0.002^**^	-0.306
PPAS_P authoritarian	64(32–102)	53.5(28–96)	2.959	0.003^**^	0.295

### Correlational Analyses

Bivariate correlations among clinical features of TTM (severity of TTM, duration of illness) and concomitant anxiety/depressive symptoms, family functioning and parenting styles were examined in the TTM group (Table 3). MGH-HPS was only positively and moderately correlated with the RCADS-CV total internalizing score (*r* =.352, *p* <.05). Duration of illness was positively and moderately correlated with the problem solving (*r* =.309, *p* <.05), affective responsiveness (*r* =.326, *p* <.05) and general family functioning (*r* =.304, *p* <.05) subscale scores of the FAD. Duration of illness was also positively and moderately correlated with the PPAS_P democratic scores (*r* =.305, *p* <.05) (Table 3).

**Table 3:** Bivariate correlations for the study variables

TTM (*n* = 50)	MGH-HPS total score	Duration of the illness	RCADS-CV /IS
MGH-HPS total score	1.00	0.094	0.011
Duration of the illness	0.094	1.00	**0.352** ^*^
RCADS-CV /IS	**0.352** ^*^	0.011	1.00
FAD- PS	0.143	**0.309** ^*^	0.009
FAD- C	0.229	0.150	0.104
FAD-R	0.070	0.123	− 0.015
FAD- AR	0.126	**0.326** ^*^	− 0.013
FAD- AI	− 0.003	− 0.164	0.030
FAD-BC	0.165	0.092	0.094
FAD- GF	0.121	**0.304** ^*^	0.001
PPAS_M democratic	− 0.143	0.260	− 0.206
PPAS_M authoritarian	0.066	0.072	0.079
PPAS_P democratic	− 0.169	**0.305** ^*^	− 0.059
PPAS_P authoritarian	0.065	0.002	− 0.023

MGH-HPS Massachusetts General Hospital Hair Pulling scale, RCADS-CV/IS revised children’s anxiety and depression scale-child version/internalizing score, FAD family assessment device, FAD-PS problem solving, FAD-C communication, FAD-R roles, FAD-AR affective responsiveness, FAD-AI affective involvement, FAD-BC behavior control, FAD- GF general functioning, PPAS-M (Perceived Paternal Attitudes Scale- Maternal), PPAS-P (Perceived Paternal Attitudes Scale- Maternal)

*p <.05

### Regression Analyses

Three separate binary logistic regression analyses were conducted to identify variables influencing the presence of TTM because of higher correlations between FAD and PPAS scores. In each regression, the following variables were added as concurrent independent variables: age, gender, SES, and total anxiety and depressive symptoms/internalizing scores of RCADS-CV. Family functioning and parenting style variables (FAD_general functioning, PPAS_M authoritarian, PPAS_P authoritarian) were entered as independent variables in separate regressions.

The results of the regression models are shown in Table 4. In the first regression, only two independent variables emerged as significant: internalizing symptoms (B = 2.767; SE = 0.557; p < .001) and FAD_general functioning (B = 0.714; SE = 0.350; p = .041). In the second and third regressions, only the following covariate emerged as significant: internalizing symptoms, respectively (B = 2.543; SE = 0.511; p < .001), (B = 2.581; SE = 0.507; p < .001). However, neither the PPAS_M authoritarian scale (B = 0.481; SE = 0.354; p = .174) nor the PPAS_P authoritarian scale (B = 0.243; SE = 0.343; p = .479) showed a significant association with TTM.

**Table 4:** Binary logistic regression analysis summary

	B	SE	Wald	*p*	OR	%95-CI	*R* ^2^
Constant	1.942	1.565	1.539	0.215	6.971	-	
Age	− 0.012	0.344	0.001	0.973	0.989	0.50-1.94	
Gender	-1.282	0.949	1.823	0.177	0.277	0.04-1.78	0.678
SES	− 0.267	0.434	0.378	0.538	0.766	0.33-1.79	
Internalizing symptoms	2.767	0.557	24.707	< 001***	15.910	5.34–47.37	
FAD_general functioning	0.714	0.350	4.178	0.041*	2.043	1.03–4.05	
Constant	1.731	1.544	1.258	0.262	5.648		
Age	0.132	0.338	0.154	0.695	1.142	0.59-2.21	
Gender	-1.119	0.967	1.339	0.247	0.327	0.05-2.17	0.661
SES	− 0.254	0.454	0.313	576	0.776	0.32-1.88	
Internalizing symptoms	2.543	0.511	24.810	< 0.001***	12.722	4.68–34.61	
PPAS_M authoritarian	0.481	0.354	1.845	0.174	1.617	0.89-3.24	
Constant	1.283	1.491	0.740	0.390	3.607		
Age	0.076	0.333	0.052	0.820	1.079	0.56-2.07	
Gender	-1.090	0.940	1.347	0.246	0.336	0.05-2.12	
SES	− 0.096	0.428	0.050	0.822	0.908	0.39 − 2.10	0.652
Internalizing symptoms	2.581	0.507	25.893	< 0.001***	13.210	4.89–35.70	
PPAS_P authoritarian	0.243	0.343	0.502	0.479	1.275	0.65 − 2.50	

Note: Independent variables were standardized using z-transformation (mean = 0, SD = 1). Coefficients represent the effect of a one standard deviation change in the independent variable on the dependent variable

^*^p <.05, **p <.01, ***p <.001

## Dıscussıon

This study investigated the family functioning and parenting styles in adolescents with TTM. The data analysis supported our hypothesis that families of adolescents with TTM reported poor family functioning, particularly in affective responsiveness, problem-solving and general functioning. General functioning was also an important predictor of TTM in adolescents. A few studies have reported impaired family functioning in pediatric TTM, particularly in affective responsiveness [16, 19, 21], consistent with the findings of this study. Adolescents who identify the maladaptive emotional responses of their parents struggle with how to interpret and respond to emotions accurately and may find it difficult to understand the emotions of others. Therefore, they may experience difficulties in understanding, accepting and effectively expressing emotions, leading to poor peer relationships, social withdrawal and the onset of internalizing symptoms [44–47]. Considering these reasons, impaired affective responsiveness in the family may serve as a risk factor for adolescent emmotion regulation difficulties. Studies have also reported that patients with TTM tend to experience negative emotions such as boredom, anger, and shame and have elevated levels of emotion regulation difficulties [48]. Although the precise nature of the relationship between TTM and impaired affective responsiveness in families remains unclear, studies assessing family functioning in patients with TTM suggest that the emotion regulation difficulties observed in these patients may overlap with impaired affective responsiveness in their families and that a significant interaction between these factors may exist. The consistent finding of impaired affective responsiveness in the families of adolescents with TTM suggests that intervening in the affective responsiveness may be an important step in planning family-focused treatment strategies.

This study also found impairment in problem-solving and general functioning in the families of adolescents with TTM. As far as we know, no studies have specifically investigated problem-solving skills in families with pediatric TTM. Conflicts are often not resolved in a timely manner in families with poor problem-solving skills. This may increase the level of stress in the family and reinforce a sense of unpredictability, which can lead to emotional dysregulation and distress in youths [46]. Recurrent and chronic family stress can negatively affect the psychosocial well-being of family members and reduce family resilience, making adolescents more vulnerable to psychiatric illnesses [49]. Studies have also reported a negative family climate characterized by high levels of criticism, increased parental feelings of anger, hostility and aggression, and excessive affective involvement in families of patients with obsessive-compulsive and related disorders (OCRD) [19, 21] and high level of negative emotional expression is negatively associated with adaptive family problem solving [50–52]. In clinical practice, it is commonly observed that patients with TTM are constantly criticized by their family members for stopping their pulling behavior, which leads to feelings of inadequacy and shame in adolescents because of their inability to stop/resist hair-pulling. At the same time, parental perception of hair pulling as a manageable behaviour rather than a psychiatric disorder may lead to failure to recognize the adolescent’s need for psychiatric support and increased parental expression of negative emotions towards the adolescent [53]. One study found that two of the main barriers to treatment for patients with TTM were the fear of being criticized by their families and low awareness about TTM [54]. Therefore, problem-solving deficits may have been detected as a result of emotional dysregulation and negative family climate combined with low awareness in families of adolescents with TTM. Problem-solving and affective responsiveness are core indicators of healthy family functioning and are closely associated with the family’s general functioning [55]. From this perspective, greater impairment in general functioning in families of adolescents with TTM seems not surprising. Given these considerations, future longitudinal studies should aim to examine the relationship between the severity and specific types of impaired family functioning and the effectiveness of family-based therapies on TTM severity and treatment adherence. Such research would contribute to the development of preventive and therapeutic family-focused interventions by identifying specific deficits in family functioning, such as affective responsiveness and problem-solving. In addition, examining the effectiveness of therapies targeting specific subdomains of family functioning in relation to the severity of dysfunction will further enhance our understanding and guide tailored treatment strategies for TTM.

This study provides the first data on authoritarian parenting styles in adolescents with TTM. Several studies have found that authoritarian parenting is consistently associated with lower self-esteem, emotion regulation deficits and socio-emotional adjustment problems in adolescents [56–58]. The emotional needs of adolescents are often ignored in families with an authoritarian parenting style. The lack of positive parental support when needed causes the adolescent to feel emotionally insecure. In addition, unidirectional hostile communication makes it difficult for adolescents to express their emotions clearly and creates a negative emotional climate at home. These factors negatively affect emotion regulation processes by increasing emotional overarousal in adolescents. On the other hand, restriction of autonomy and frequent use of punitive strategies disrupt the development of a healthy sense of self-worth and identity, which may increase an adolescent’s socio-emotional adjustment problems [59]. The research has also shown similar effects of maternal and paternal parenting styles on adolescents’ psychosocial well-being, and parents tend to use similar parenting styles. The same research suggests that people tend to interact more often with those who have similar parenting styles, and their perspectives on parenting become more similar through observation and shared experiences [60–62]. On the other hand, when comorbid anxiety/depression symptoms were included in the logistic regression model, authoritarian parenting style ceased to be a significant predictor. For all of these reasons, it can be concluded that the authoritarian parenting style is an important predisposing factor that negative impacts on the overall socio-emotional adjustment of adolescents and paves the way for the development of many mental health disorders, including TTM. Given the central role of parental attitudes in shaping the socio-emotional development and psychological resilience of adolescents, the assessment of parenting styles is a critical component of the psychiatric assessment of adolescents presenting to mental health clinics. Parents’ awareness of their own attitudes will increase the effectiveness of the treatment process by facilitating what is necessary for the adolescent’s well-being.

This study found a positive and moderately significant relationship between the severity of TTM and comorbid anxiety/depression symptoms, and these symptoms were also demonstrated to be one of the predictors of TTM in adolescents. Consistent with our findings, numerous studies have shown that one of the most important predictors of TTM severity is the presence of comorbid anxiety/depression symptoms [63–66]. Comorbid anxiety/depression symptoms increase the functional impairment in adolescents with TTM, resulting in reduced treatment compliance and response, which has a negative impact on the clinical course of TTM. It has also been reported that there is a significant association between prolonged time from the onset of hair-pulling to psychiatric admission and the development of comorbid psychiatric symptoms [63, 64, 67]. The perception of TTM as a harmless and manageable habit, combined with the social isolation and avoidance caused by hair pulling behavior, delays patients from seeking help from mental health professionals. These patients usually seek professional help when comorbid psychiatric disorders occur [37, 66, 67]. Therefore, a high prevalence of comorbid anxiety/depression symptoms in adolescents presenting to clinics with complaints of hair pulling and diagnosed with TTM is an expected finding. However, the relationship between TTM and comorbid anxiety/depression symptoms is bidirectional [64]. The negative outcomes of TTM, such as embarrassment, negative social evaluation and lower self-esteem, have the potential to exacerbate the associated anxiety and depression symptoms. In contrast, considering adolescence as a critical period for internalizing disorders, increased levels of stress, unhappiness, hopelessness and numbness may act as catalysts for the onset or exacerbation of TTM [64]. For these reasons, comprehensive assessment and treatment of comorbid psychiatric symptoms in adolescents with TTM is essential in the effective management of TTM. Furthermore, longitudinal studies with large sample sizes are required to have a clear understanding of the bidirectional relationship between TTM and comorbid anxiety/depression symptoms.

In this study, there were no statistically significant relationships between the severity of TTM and any domain of family functioning and parenting styles, consistent with the results of Peris et al. [20]. There are several possible explanations for this finding. Although impaired family functioning and authoritarian parenting styles are more common and related to the onset of TTM symptoms, psychiatric comorbidities may be essential in determining the chronicity or exacerbation of symptoms after the onset of the disorder. Secondly, while hair-pulling symptoms may fluctuate over time, parenting styles and family functioning remain stable [21, 68]. This can make it difficult to clearly define the effect of family environment variables on the severity of TTM. Finally, using a self-report scale that evaluates the severity of TTM for only the most recent week may have prevented us from getting realistic results of the severity of TTM, which is one of the limitations of our study.

This study also found positive and moderately significant correlations between the duration of illness and impairments in family functioning, namely affective responsiveness, problem-solving and general family functioning. Although there is no related study, these families with a lack of constructive and harmonious affective responsiveness and practical problem-solving skills may tend to consult mental health professionals when the adolescent shows significant impairment in functioning or when comorbid psychiatric symptoms exacerbate the illness. Moreover, impaired family functioning reduces the adolescent’s compliance with treatment and may make it difficult to maintain therapeutic progress. From another perspective, TTM has a profound impact not only on the individual, but also on the overall family functioning. The perception of a ‘loss of health’ often triggers intense emotional reactions among family members, including helplessness, guilt and disappointment, which can be further amplified if these behaviors persist over time. The shame and guilt associated with hair-pulling behavior, and the prolonged preoccupation with this behavior, can lead to social isolation for both patients and their families. In addition, the waxing and waning nature of TTM, with its unpredictable clinical course, can exacerbate family stress and impose a substantial financial burden on the family due to ongoing medical expenses [69, 70]. The direction of the relationship between family functioning and duration of illness remains unclear, highlighting the need for further research to elucidate the complexities of their potential bidirectional interaction.

Despite the important findings of this study, the results must be interpreted in light of some limitations. As this study had a cross-sectional design, possible bidirectional relationships between the variables studied could not be determined. In addition, the cross-sectional design makes it impossible to establish a causal relationship between the variables. Although the sample size is acceptable compared to the existing literature, future studies with a larger sample size may contribute to more precisely determining the relationships between family environment and TTM. The results were controlled for additional factors such as family structure and SES that may influence family functioning. However, it is important to recognize that family functioning is a complex construct influenced by various variables such as life stressors, marital adjustment, overall psychiatric burden, the family’s socio-cultural level and the family’s social support systems. Therefore, future research should address this issue.

### Summary

In conclusion, family functioning and parenting styles represent an important area for further research in understanding the etiology and developing effective treatment strategies for TTM. This study examines family functioning and parenting styles in adolescents with TTM compared to healthy controls and explores the relationships between family functioning, parenting styles, and the clinical characteristics of TTM. Findings reveal significantly higher levels of comorbid anxiety and depressive symptoms in adolescents with TTM, which emerged as the strongest predictors of the severity of the TTM symptoms. While parents of adolescents with TTM reported greater impairments in affective responsiveness, problem-solving, and general family functioning, adolescents with TTM perceived both parents as significantly more authoritarian. In addition, the duration of illness was positively and moderately correlated with the impairment in affective responsiveness, problem-solving and general family functioning. Studies with large samples and longitudinal designs that take into account genetic, familial, psychosocial and psychocultural factors that influence family functioning will contribute to our understanding of the nature and direction of the relationship between family functioning, parenting styles and TTM.

## Data Availability

No datasets were generated or analysed during the current study.
